# Testing Luminescence Dating Methods for Small Samples from Very Young Fluvial Deposits

**DOI:** 10.3390/mps2040090

**Published:** 2019-12-06

**Authors:** Joel Q. G. Spencer, Sébastien Huot, Allen W. Archer, Marcellus M. Caldas

**Affiliations:** 1 Department of Geology, Kansas State University, Manhattan, KS 66506, USA; aarcher@ksu.edu; 2 Illinois State Geological Survey, Prairie Research Institute, Champaign, IL 61820, USA; shuot@illinois.edu; 3 Department of Geography and Geospatial Sciences, Kansas State University, Manhattan, KS 66506, USA; caldasma@ksu.edu

**Keywords:** Amazon, Rio Tapajós, quartz OSL dating, small samples, very high specific sensitivity, very young fluvial deposits

## Abstract

The impetus behind this study is to understand the sedimentological dynamics of very young fluvial systems in the Amazon River catchment and relate these to land use change and modern analogue studies of tidal rhythmites in the geologic record. Initial quartz optically stimulated luminescence (OSL) dating feasibility studies have concentrated on spit and bar deposits in the Rio Tapajós. Many of these features have an appearance of freshly deposited pristine sand, and these observations and information from anecdotal evidence and LandSat imagery suggest an apparent decadal stability. The characteristics of OSL from small (~5 cm) sub-samples from ~65 cm by ~2 cm diameter vertical cores are quite remarkable. Signals from medium-sized aliquots (5 mm diameter) exhibit very high specific luminescence sensitivity, have excellent dose recovery and recycling, essentially independent of preheat, and show minimal heat transfer even at the highest preheats. These characteristics enable measurement of very small signals with reasonable precision and, using modified single-aliquot regenerative-dose (SAR) approaches, equivalent doses as low as ~4 mGy can be obtained. Significant recuperation is observed for samples from two of the study sites and, in these instances, either the acceptance threshold was increased or growth curves were forced through the origin; recuperation is considered most likely to be a measurement artefact given the very small size of natural signals. Dose rates calculated from combined inductively coupled plasma mass spectrometry/inductively coupled plasma optical emission spectrometry (ICP-MS/ICP-OES) and high-resolution gamma spectrometry range from ~0.3 to 0.5 mGya^−1^, and OSL ages for features so far investigated range from 13 to 34 years to several 100 years. Sampled sands are rich in quartz and yields of 212–250 μm or 250–310 μm grains indicate high-resolution sampling at 1–2 cm intervals is possible. Despite the use of medium-sized aliquots to ensure the recovery of very dim natural OSL signals, these results demonstrate the potential of OSL for studying very young active fluvial processes in these settings.

## 1. Introduction

### 1.1. Background

An important facet of the development of a geochronological technique is the investigation of potential age range. Much recent work in the luminescence field has focused on maximum achievable ages using high-temperature post-infrared infrared (pIRIR) signals from feldspars [1,2]. In contrast for quartz optically stimulated luminescence (OSL), the more efficient signal resetting coupled with environments where grain reworking is evident make it well suited to assessment of minimum achievable age. Notable examples are studies of young fluvial deposits [3,4,5,6] and dunes [7,8,9,10,11].

Regarding the application of OSL dating to fluvial sediments in the Amazon region, a number of studies have used the technique to try to constrain the origin and development of the drainage system, documenting Mid–Late Pleistocene ages [12,13,14], and OSL analyses have also been carried out to investigate the Late Pleistocene to Holocene development of fluvial bars [15].

The impetus behind this work was to investigate the feasibility of optically stimulated luminescence (OSL) dating of very young fluvial and shoreline landforms in the Amazon River catchment. The ultimate goal of the study is to use OSL to help understand the sedimentological dynamics of fluvial systems in the Amazon. This has relevance to the important issue of the anthropogenic effect of decades of land use and land cover change on the Amazon biome [16,17,18], that has impacted the stock of carbon and biodiversity [19,20] and resulted in erosion in many areas of the basin including along the rivers [21]. Furthermore, the Amazon is subject to significant marine tides, which propagate inland 1000 km from the mouth region, and OSL data have the potential to contribute to depositional models for modern analogues of ancient tidal rhythmites [22]. Initial OSL dating feasibility studies have concentrated on fluvial/shoreline features in the Rio Tapajós.

### 1.2. Geologic Setting

**Figure 1:**
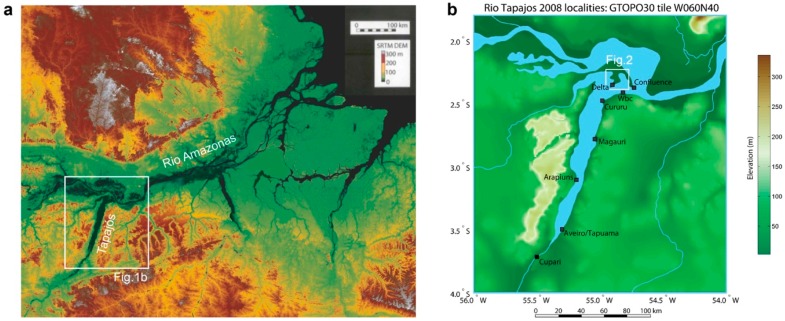
(**a**) Shuttle Radar Topography Mission Digital Elevation Model (SRTM DEM) image [26]. Box indicates study area in lower ~160 km stretch of the Rio Tapajós. (**b**) Detail of study area (USGS Global 30 Arc-Second Elevation data, GTOPO30, [27]) indicating sampling sites (black squares). The work described here investigated samples from Cupari, Tapuama and Arapiuns.

**Figure 2:**
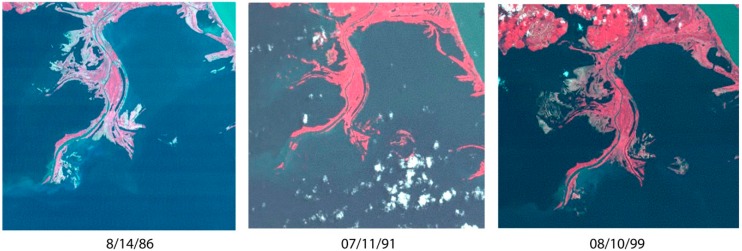
Historic LandSat imagery of bird’s foot delta, northern Tapajós, indicates only minor morphological changes over the past four decades.

The Rio Tapajós is a major river system draining the Amazon basin, running ~1930 km from the Mato Grosso plateau (14°25′ S) north to the confluence with the Rio Amazonas at Santarém (2°25′ S) (Figure 1a). In the last ~160 km the Tapajós widens to 6–14 km, deepens considerably, and forms a ria (flooded river valley) (Figure 1b). To the south, deposits of pristine quartz-rich sand line the banks of the ria. These sands are primarily sourced by Cretaceous sandstone bedrock [23,24] that forms prominent bluffs as high as 90–120 m. Because of prevailing, equatorial tradewinds, out-of-phase peak discharge between Amazon mainstem (May–July) and Tapajós (March–May) [25], and potential tidal influence [24], the spits and bars exhibit an unusual pattern of upstream progradation. LandSat imagery indicates depositional systems have undergone only minor morphological changes in four decades (Figure 2). On the Amazonas mainstem at Santarém, a ~6 m seasonal oscillation of river level is documented [22]. Examples of both subaerially exposed and subaqueous spits and bars were identified in the Tapajós during fieldwork; the range in seasonal oscillation of the Tapajós is not as well documented but has been reported to be a similar order to the mainstem at ~5 m [24].

The drainage basin of the Tapajós is covered with dense rainforests on highly weathered, ancient shields, resulting in a clearwater river. Conversely, the mainstem of the Amazon has very high suspended sediment loads. Floodplain deposition along the Amazon has kept pace with Holocene rise in base level. Along the Tapajós, however, lack of sediment has resulted in a ria that is partially dammed along the Tapajós-Amazon confluence. The waters within these two disparate types of rivers maintain individual identity downstream of this confluence. A zone of mixing, very similar to the “meeting of the waters” at Manaus, occurs along the riverfront at Santarém.

## 2. Study Area and Sampling

Our study focused on shoreline features (spits, bars and dunes) that were accessed by boat and speedboat. The study area and sampling localities are shown in Figure 1b. Sands were sampled with a vertical push corer, with black spray-painted plastic sleeve inserts, allowing cores of ~65 cm in length by ~2 cm in diameter to be collected. The painted sleeves were examined carefully for complete paint coverage and tested to ensure bright white light was not visible through the painted exterior. Empirical luminescence tests of the light-tightness of the sleeves were not considered necessary but, as an added precaution, all core samples were promptly capped and immediately wrapped in thick black plastic when removed from the corer.

The work described in this study investigated core samples from three southerly sampling localities at Cupari, Tapuama and Arapiuns. At Cupari, duplicate samples (CUP-030808-01 and CUP-030808-02) separated by ~2–3 m were collected from a densely vegetated sand bar ~150 m from the riverbank. At the Tapuama locality, two samples were collected from a spit. The first (TAP-030808-03) from the unvegetated southerly distal end and the second (TAP-030808-04) from sands a few meters within the vegetated proximal end. At Arapiuns, a single sample (ARA-040808-05) was collected from the crest of a shoreline Aeolian dune directly behind a sandy beach. Sampling was conducted in the month of August, when fluvial discharge is roughly half-way between maximum (March–May) and minimum (September–December) flow periods [24]. The sampled cores were collected above the observable river water level, and probably above reach of capillary fringe influence (~0.2–0.3 m in sands [28]). Given the seasonal oscillation of the Tapajós, even if ~5 m (Section 1.2, [24]) is an overestimate, we anticipate the sampling sites oscillate between subaerial and subaqueous fluvial landforms. The altitude of all sampling sites was recorded as ~10 m above sea level with a hand-held GPS.

## 3. Luminescence Studies

### 3.1. Sample Preparation

Preparation procedures to produce 212–250 μm or 250–310 μm quartz grains for OSL analyses were carried out under low-intensity red safe lighting. Sediment at a core depth of ~63–65 cm was removed from the end of each core to exclude the possibility of analyzing grains that had been exposed to daylight during sample retrieval. The next ~5 cm from a core depth of ~58–63 cm was prepared for luminescence analyses. Standard preparation steps [29] included dry sieving, 10% HCl and 30% H_2_O_2_ pre-treatments to remove carbonates and organic matter, respectively, separation of heavy minerals (>2.70 gcm^−3^) with lithium metatungstate (LMT) heavy liquid, and treating with 48% HF for 40 min to dissolve feldspar minerals and etch the surface of quartz grains to minimize luminescence due to ionization from external alpha particles. Initial test measurements indicated the dimmest natural OSL signals could only be recovered with use of medium-sized (5 mm diameter) aliquots. Potentially such small signals could be observed if a large number (e.g., ~100) of small (e.g., 1 mm) multi-grain aliquots were analyzed but given practical limitations of machine time we prepared medium-sized aliquots for all samples analyzed. Monolayers (5 mm circles; ~280 grains of 250–310 μm, and ~420 grains of 212–250 μm) of quartz grains were dispensed onto ~9.7 mm diameter stainless steel discs using silicone oil and a spray template.

The sediment at a core depth of ~63–65 cm was used for inductively coupled plasma mass spectrometry / inductively coupled plasma optical emission spectrometry (ICP-MS/ICP-OES) measurements and at a core depth of ~53–58 cm for high-resolution gamma spectrometry. After drying, these samples were pulverized in a Shatterbox ring and puck mill before sending for analysis.

### 3.2. Measurements

OSL measurements were carried out using a Risø TL/OSL-DA-20 reader [30], with optical stimulation of quartz provided by an array of blue light (470 nm, FWHM 20 nm) diodes, optical stimulation of feldspar with infrared (870 nm, FWHM 40 nm) diodes, a calibrated ^90^Sr/^90^Y beta source (~0.16 Gys^−1^) to administer laboratory radiation doses, and a heating stage for thermal stabilization. All luminescence signals were detected in the ultraviolet (peak transmission ~340 nm) using 7.5 mm of Hoya U-340 filter with an EMI 9235QB photomultiplier tube.

Determination of the equivalent dose (*D*_e_) was carried out using a single-aliquot regenerative-dose (SAR) protocol [31,32,33] with modifications. Continuous power or continuous wave OSL (CW-OSL) was conducted in all measurements. We routinely utilize post-infrared optically stimulated luminescence (post-IR OSL) measurement approaches [29,34,35,36,37,38,39,40], which in certain instances has been shown to improve dose recovery results even if infrared signals are negligible or absent [29]. Post-IR OSL was used to measure the luminescence from the quartz grains in this study. This procedure removes charge sensitive to infrared stimulation, commonly associated with remnant feldspathic minerals, before measuring OSL from the quartz grains. The post-IR OSL measurement comprised 40 s infrared-stimulated luminescence (IRSL) at ~117.9 mWcm^−2^ (22 Vishay TSFF5200 IR led’s at 90% power) at a sample temperature of 50 °C, followed by 40 s OSL at 38.7 mWcm^−2^ (28 Nichia NSPB500S blue led’s at 90% power) at a sample temperature of 125 °C. After measurement of the natural OSL in the first SAR cycle, regenerative doses in subsequent cycles were approximately 0.8, 1.6, 2.4, 0, and 0.8 Gy. The test dose administered for sensitivity correction was typically ~1.6 Gy, exceeding typical *D*_e_ values by a factor of ~10 to 400 (consistent with data from [7]). Test doses were heated to 160 °C prior to measurement. A hot bleach measurement of 40 s OSL at 280 °C was incorporated at the end of each SAR cycle [32].

**Figure 3:**
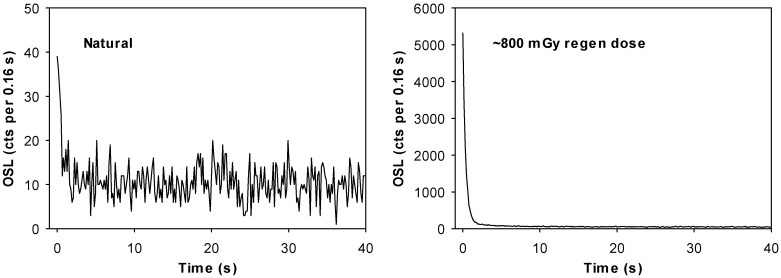
Typical optically stimulated luminescence (OSL) signals for an aliquot from sample Tapuama (TAP)-030808-03.

All natural IRSL signals appeared negligible but natural OSL signals (Figure 3) were also very dim. There was little observable scaling in size of IRSL at the regenerative dose level suggesting IR contamination may not be a problem. However, for such small OSL signals, negligible IRSL may still be a source of overestimation and requires careful assessment if a post-IR protocol is not used.

For all measurements we compared two approaches to define the net OSL signal: (1) late background subtraction where the signal was defined as the initial 0.8 s integral with subtraction of the final 8 s integral [31], and (2) early background subtraction with the same initial 0.8 s integral with subtraction of the following 0.8 to 2.72 s integral. The latter method has been assessed to optimize the contribution from the fast component [41]. The *D*_e_ value was estimated by interpolation of the natural OSL with a best-fit linear or saturating exponential curve fitted to regenerative OSL data. Uncertainty in *D*_e_ was estimated by combining error from counting statistics for the natural OSL, curve fitting, and instrumental systematic uncertainty [42].

Dose-rate measurements were conducted using the core portions described in Section 3.1. High-resolution gamma spectrometry was performed using a small-sample 2 g well geometry for assessment of U and Th. With the use of such a small sample for gamma spectrometry, a homogenous medium is assumed for accurate assessment of the radioactivity within a 30 cm radius sphere; the fluvial sand samples studied here are of uniform composition with well-sorted grain sizes, and thus a homogeneous medium is a good approximation. Li-metaborate fusion ICP-OES and ICP-MS were performed for K and Rb, respectively. These data were converted to annual dose rate using conversion factors [43]. Calculated beta dose was corrected using attenuation factors for grain size and HF etching (described in detail in [44] and references therein). In the absence of detailed imagery or documented evidence of the nature of subaerial-to-subaqueous cyclicity at the Tapuama and Cupari sites, attenuation of dose rate via moisture conditions over the burial time of the samples was calculated by using present day field moisture content with a maximum absolute error of 5% to allow for past changes. The dose rate from the ionizing cosmic ray component was calculated following [45]. For the purposes of this feasibility study, a constant overburden depth was assumed; we deliberately chose the deepest part of the cores for our sample selection in an attempt to minimize shallow gamma and hard cosmic corrections [46]. Finally, an estimate of an internal dose rate of 0.01 ± 0.002 mGya^−1^ [47] was incorporated into total dose-rate assessment.

### 3.3. Luminescence Characteristics

#### 3.3.1. Specific Sensitivity of OSL Signals

An important consideration with dating features on an annual to decadal timescale is whether the luminescence is sufficiently sensitive to enable measurement of very small radiation doses. Quartz from the Tapajós shows weak natural OSL signals but very high specific sensitivity in response to a small regenerative dose (Figure 3). In Section 1.2 above, we suggest that the primary source of sand in the Tapajós is from Cretaceous sandstone bedrock that forms prominent bluffs. Given the high sensitivity of the quartz, the interplay of sources from Paleozoic [23] or even Paleoproterozoic [48] igneous and metamorphic rocks from the Amazon Craton must also be considered.

#### 3.3.2. Effect of Preheating

**Figure 4:**
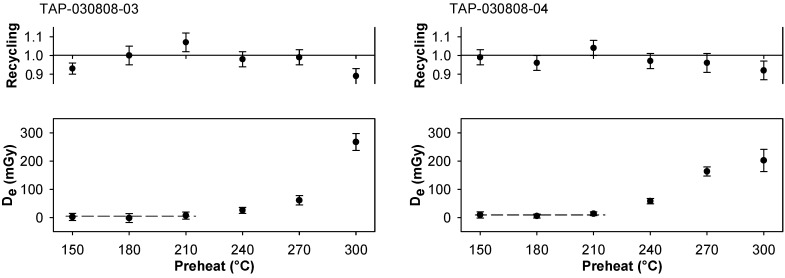
Determination of the equivalent dose (*D*_e_) plateau test results on samples from Tapuama.

**Figure 5:**
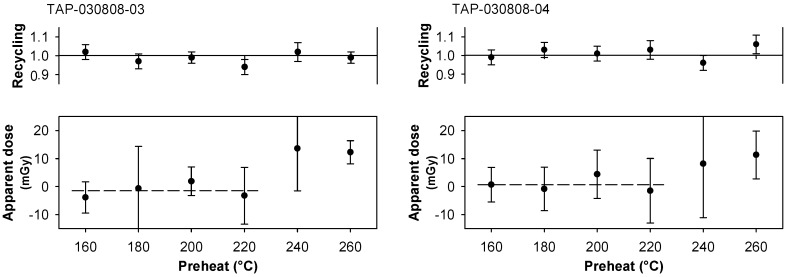
Thermal transfer test results on samples from Tapuama.

The importance of investigating the influence of preheating for very young samples has been emphasized in previous studies [7]. We carried out *D*_e_ plateau tests and thermal transfer tests on all samples investigated; the latter tests were carried out using an optical bleach at ambient temperature (40 s OSL, 4000 s pause, 40 s OSL; [7]). For the samples from Tapuama, these data indicate a low temperature *D*_e_ plateau region below 210 °C (Figure 4), and minimal thermal transfer in a similar temperature band (Figure 5). The samples from Cupari and Arapiuns showed similar characteristics. A plateau in *D*_e_ data is a possible indication of complete resetting but, given the medium aliquots utilized in this work, such an interpretation must be treated with some caution.

#### 3.3.3. Dose Recovery Tests

**Figure 6:**
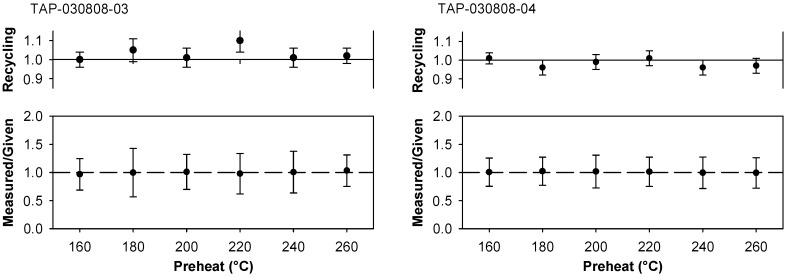
Dose recovery test data on samples from Tapuama.

**Figure 7:**
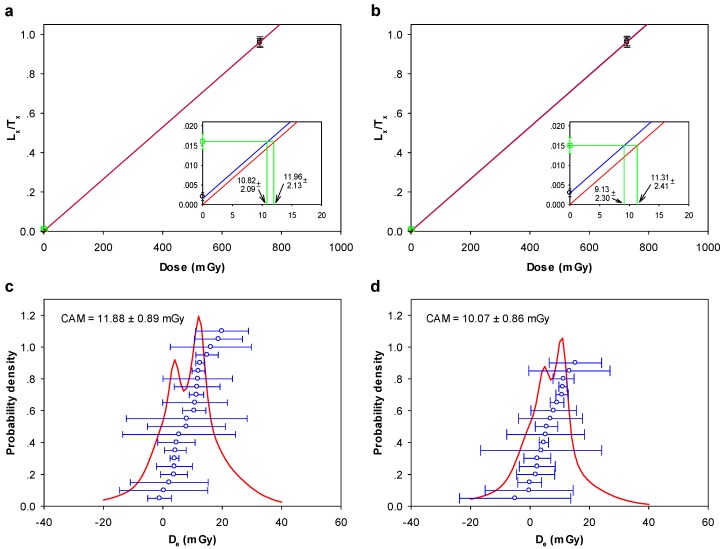
(**a**) and (**b**): Examples of growth curves of sensitivity-corrected OSL (L_x_/T_x_) with regenerative dose for two aliquots of Arapiuns (ARA)-040808-05. Only growth to first regenerative recycled point is shown, with linear fit corresponding to all regenerative dose points. Inset figures show details of interpolation with the natural OSL in the first 20 mGy of the growth curve. Blue line is fit to L_x_/T_x_ value for zero regenerative dose, red line is fit forced through the origin, and green symbol and lines are interpolation of natural OSL to both growth curve fits. Corresponding *D*_e_ values for both fits are shown. Recuperation in (**a**) and (**b**) is 9.7 ± 5.4% and 19.5 ± 7.2%, respectively. (**c**) and (**d**): Distribution of *D*_e_ data when growth curves are forced through the origin (**c**), compared to growth curve fitting to zero regenerative dose (**d**). In (**d**), acceptance threshold for recuperation was set at 35%, with 18 of 22 aliquots accepted.

Dose recovery tests with preheat variation [29] were carried out on all the samples investigated. Test results that typify the behavior of all samples are shown in Figure 6 for TAP-030808-03 and TAP-030808-04. The given dose was ~1.6 Gy (10 s beta exposure). This value was considerably larger than the *D*_e_ values indicated in Figure 4 and Figure 7, and Table 1, but was considered prudent because the offset time [49] for our source had not been determined, and even doses administered for 1 s of source exposure would exceed *D*_e_ by a factor of ~40 for youngest samples. An implication of using a relatively large given dose is that evidence of the thermal transfer signal above ~220 °C is masked (Figure 6). Despite this, measured-to-given ratios for all samples were close to unity and imply the first sensitivity measurement is appropriate to the preceding natural OSL [32,33], and support choice of preheat (see Table 1) from *D*_e_ plateau tests and thermal transfer tests.

### 3.4. Towards OSL Dating of Multi-Grain Quartz Aliquots

A summary of the OSL analysis is given in Table 1. *D*_e_ data, calculated using late background subtraction, were indistinguishable from those data analyzed using early background subtraction. An important observation is that recuperation measured during the *D*_e_ SAR cycle is significant for the quartz from Arapiuns (Figure 7a,b) and Tapuama. For the Arapiuns sample (ARA-040808-05), we calculate a similar *D*_e_ result when the growth curves are forced through the origin (Figure 7c; all aliquots accepted) compared to when the acceptance threshold for recuperation was set at 35% to achieve a satisfactory *D*_e_ dataset (Figure 7d; 18 of 22 aliquots accepted); for Tapuama (TAP-030808-03 and TAP-030808-04), recuperation was much more significant and as a consequence all growth curves were forced through the origin to obtain *D*_e_ values. High recuperation is somewhat surprising given the minimal thermal transfer for preheats <~220 °C (Figure 5), and the likelihood of numerous bleaching events occurring in these shoreline environments which have been linked to substantially reduced recuperation effect [50]. Given that the values measured are unusually high (e.g., for Tapuama aliquots, recuperation exceeds L_n_/T_n_ by a factor ranging from ~1 to ~60), we suspect that the majority of the recuperation signal recorded could be a measurement artefact due to the comparatively large regenerative doses (lowest beta dose was ~800 mGy) used compared to the measured *D*_e_ values (Table 1). Furthermore, if for example the *D*_e_ values were ~400 mGy, then the majority would be accepted below the 5% threshold level. Future work will investigate whether there is a systematic dose-dependent effect on the size of the recuperation signal.

Over-dispersion (*σ*_b_; [51]) values of 0% for the Arapiuns and Tapuama samples indicate the OSL signals were completely reset, although the true extent of resetting may not be revealed due to the medium-sized aliquots measured. For these samples the final *D*_e_ was calculated using the central age model (CAM) [52]. The second of the Cupari samples, CUP-030808-02, had a moderate over-dispersion value of 13.3%, but was also well suited to a CAM analysis. CUP-030808-01 was the only sample with a lower quartz yield and, subsequent to *D*_e_ plateau, thermal transfer and dose recovery tests, only 10 aliquots were available for *D*_e_ analysis. Over-dispersion for this sample was higher at 20.1% but, despite the low number of aliquots a minimum age model (MAM; [52]), analysis returned a result with a reasonable number of significant aliquots contributing to the MAM result (*p*-value = 0.333).

The OSL age for sample ARA-040808-05, from the shoreline dune feature at Arapiuns, is 24 ± 3 a from 2009. For Tapuama, the sample from the unvegetated distal end of the spit, TAP-030808-03, is 13 ± 5 a, and from the sands within the vegetated proximal end of the spit, TAP-030808-04, is 34 ± 8 a. Although we lack direct independent dating evidence, these are plausible ages for the Tapuama samples, with the sands from the unvegetated distal end of the spit of younger depositional age than the sands in the vegetated proximal end of the spit. Vegetation adds stability to sediments via root networks increasing cohesive strength, and by grasses, shrubs, and trees increasing surface roughness and dissipating some energy of wind or water; together, these lower the effectiveness of erosion by wind or water. The result for TAP-030808-04 is supportive of apparent decadal stability of other vegetated landforms (cf. Figure 2), whereas TAP-030808-03 suggests continual reworking and redeposition occurs in more active zones of the spit. The duplicated samples from the densely vegetated bar at Cupari have significantly older OSL ages of 324 ± 29 a and 557 ± 35 a; these ages in the 100s-of-years range are more consistent with youngest ages from other OSL studies of Tapajós sand bars [15]. Although the Cupari bar seems likely to be an older feature, we suspect that the discordance in the two ages may in part be related to poor resetting that is not apparent because of the large aliquots measured. For these samples the natural OSL is sufficiently large that smaller aliquots, or potentially single grains, could be measured to investigate this age discordance. ^210^Pb data from a series of channel bottom cores from the Tapajós indicate sedimentation rates of 0.2–0.7 cmyr^−1^ in the upper stretch of the ria (consistent with the sampling localities in the work described here), and 0.2–1.9 cmyr^−1^ sampled across the entire ria [25]. If we make the assumption that these values represent sedimentation rates not only for clays, silts, and sands in the channel bottom but also for sands in shoreline features, we derive an age range of ~30–300 years (for 60 cm depth assuming uniform linear deposition) similar to the age range indicated from this OSL study.

This study demonstrates the potential of OSL to determine depositional age of very young fluvial landforms in the Rio Tapajós. Importantly it has revealed how future experimental approaches should be modified in the following ways: (1) use of ultra-low-dose beta source (and assessment of possible dose-dependency of recuperation); (2) optimization of aliquot size or single grain analyzes (including assessment of F-statistic [53] and un-logged age model approaches [6]); and (3) considering the seasonal river-level oscillation and extremely low external dose rates, careful assessment of water content fluctuation, accurate measurement of internal dose rates, and modelling of gamma and cosmic dose rates [46]. Given the nature of this study as one of feasibility of OSL approaches on a small selection of samples, it follows that geomorphological interpretation is somewhat speculative and should be limited. We propose future work with detailed stratigraphic and lateral sampling strategies which, in combination with differential remotely sensed imagery and hydrological data, will provide a sensitive monitor of how fluvial landforms change in response to land cover and land use change (including planned dam projects) and modern analogue data for depositional models of ancient tidal rhythmites.

The landforms investigated in this study were all quartz rich, but quartz yield was dependent on grain size distribution. For the ~5 cm core samples from Cupari, the majority of the sample was >310 μm, and quartz yields for sieve fractions <310 μm were correspondingly low. Conversely, ~5 cm core samples from Arapiuns and Tapuama had high quartz yields in the 250–310 μm and 212–250 μm sieve fractions, respectively. This indicates that in certain localities, a 1–2 cm sampling resolution may be possible.

## 4. Conclusions

In this study, we have investigated the feasibility of OSL dating of small samples of very young quartz collected from bar, spit and dune shoreline features along the Rio Tapajós, Brazilian Amazon. Five samples were collected from three study sites in ~65 cm by ~2 cm diameter vertical cores. Small subsamples from ~58–63 cm core depth were analyzed. The measured OSL signals exhibit very high specific luminescence sensitivity, have excellent dose recovery and recycling, essentially independent of preheat, show minimal thermal transfer below ~220 °C, and have a low temperature *D*_e_ plateau in a similar temperature band. Significant recuperation is observed for samples from two of the study sites but, given the minimal thermal transfer and likely numerous bleaching–burial cycles, we propose that the recuperation is possibly a measurement artifact due to the relatively high regenerative and test doses compared to the natural dose. Preliminary ages of features so far investigated range from 13 to 34 to several 100 years. Sampled sands are rich in quartz, and yields of 212–250 μm and 250–310 μm grains indicate high-resolution sampling is possible. These results demonstrate the potential of OSL for studying very young active fluvial processes in these settings.

## Figures and Tables

**Table 1:** Summary of OSL data.

Sample ^a^	Lat., Long. (°S, °W)	n ^b^	Preheat ^c^ (°C)	Recuperation threshold ^d^	*σ*_b, *D*e_^e^ (%)	*D*_e_^f^ (mGy)	Dose Rate (mGya^−1^)	Age ^g^ (a)
ARA-040808-05	3°6′3″	18 (22)	220	35%	0	10.1 ± 0.86 ^c^	0.42 ± 0.04	24 ± 3
	55°13′39″							
TAP-030808-03	3°29′33″	12 (12)	200	Origin fit	0	4.10 ± 1.56 ^c^	0.31 ± 0.02	13 ± 5
	55°15′41″							
TAP-030808-04	3°29′31″	12 (12)	200	Origin fit	0	12.2 ± 2.83 ^c^	0.36 ± 0.03	34 ± 8
	55°15′39″							
CUP-030808-01	3°42′44″	10 (10)	200	5%	20.1	147 ± 9.80 ^m^	0.46 ± 0.03	324 ± 29
	55°23′45″							
CUP-030808-02	3°42′44″	23 (23)	220	5%	13.3	261 ± 7.48 ^c^	0.47 ± 0.03	557 ± 35
	55°23′45″							

^a^ Samples are arranged from northerly-to-southerly sampling localities; further details in Section 2. ^b^ Number of aliquots accepted for *D*_e_ analysis (figures in parentheses are total number measured). Sample Cupari CUP-080308-01 had a lower quartz yield and only ten aliquots were measured for *D*_e_ analysis. ^c^ Ten-second preheat for *D*_e_ measurement chosen from a combination of preheat plateau, thermal transfer and dose recovery test data; cutheat was 160 °C for all measurements. ^d^ Individual *D*_e_ values were accepted if recuperation was below the specified threshold; for the Tapuama samples the growth curves were forced through the origin. ^e^ Over-dispersion in *D*_e_ data. ^f^ Superscript ‘c’ indicates central age model (CAM) result; superscript ‘m’ indicates minimum age model (MAM) result. ^g^ OSL ages quoted are in years (a) from 2009 with 1-sigma uncertainty.
